# Extension of lifespan by epicatechin, halofuginone and mitoglitazone in male but not female genetically heterogeneous mice

**DOI:** 10.1007/s11357-025-01881-6

**Published:** 2025-09-19

**Authors:** Randy Strong, James F. Nelson, Molly A. Bogue, Jerry R. Colca, Martin Denzel, Vivian Diaz, Brian N. Finck, Vadim N. Gladyshev, Steve Horvath, Nisi Jiang, Tracy Keller, Rolf F. Kletzien, Ron Korstanje, Navasuja Kumar, Christiaan Leeuwenburgh, Elizabeth Fernandez, Andrzej Galecki, Brett Ginsburg, Melissa Han, Catherine Kaczorowski, Scott Leiser, Marisa Lopez-Cruzan, Ken Raj, Peter C. Reifsnyder, Nadia A. Rosenthal, Susanna Rosi, Anastasia Shindyapina, Peter Stacpoole, Adam B. Salmon, Alexander Tyshkovskiy, Peter Walter, Malcolm Whitman, Richard A. Miller, David E. Harrison

**Affiliations:** 1 Geriatric Research, Education and Clinical Center and Research Service, Department of Pharmacology, Barshop Institute for Longevity and Aging Studies, South Texas Veterans Health Care System, The University of Texas Health Science Center at San Antonio, San Antonio, TX 78229 USA; 2 Department of Pathology and Geriatrics Center, University of Michigan, Ann Arbor, MI 48109 USA; 3 Department of Cellular and Integrative Physiology and Barshop Institute for Longevity and Aging Studies, The University of Texas Health Science Center at San Antonio, San Antonio, TX 78229 USA; 4 Geriatric Research, Education and Clinical Center, Department of Molecular Medicine, Barshop Institute for Longevity and Aging Studies, South Texas Veterans Health Care System, University of Texas Health Science Center at San Antonio, San Antonio, TX 78229 USA; 5 Departments of Internal Medicine and Biostatistics, School of Medicine and School of Public Health, University of Michigan, Ann Arbor, MI 48109-2200 USA; 6 Department of Psychiatry, University of Texas Health Science Center at San Antonio, San Antonio, TX 78229 USA; 7 The Jackson Laboratory, Bar Harbor, ME 04609 USA; 8 Cirius Therapeutics, Inc, Kalamazoo, MI USA; 9 Max Planck Institute for Biology of Ageing, Joseph-Stelzmann-Str. 9B, 50931 Cologne, Germany; 10 Division of Nutritional Sciences and Obesity Medicine, Department of Medicine, Washington University School of Medicine, St. Louis, MO 63110 USA; 11 Human Genetics, David Geffen School of Medicine, UCLA. Epigenetic Clock Development Foundation, Los Angeles, CA 90095 USA; 12 Department of Biomedical Sciences, Metabolic Solutions Development Company (MSDC), Western Michigan University School of Medicine, KalamazooGrand Rapids, MI 49007.MI 49546 USA; 13 Institute On Aging, Department of Physiology and Aging, College of Medicine, University of Florida, Gainesville, FL 32611 USA; 14 Department of Medicine. Division of Endocrinology, Diabetes and Metabolism, University of Florida. , 1600 SW Archer Road Gainesville, Florida, 32610-0226 USA; 15 Department of Developmental Biology, Harvard School of Dental Medicine, Boston, MA 02115 USA; 16 Altos Labs, Bay Area Institute, 13-Island Dr, Redwood City, CA 94065 USA; 17 Dept of Biochemistry & Biophysics, Howard Hughes Medical Institute, University of California San Francisco, MC2200. 600 16Th St, N316, San Francisco, CA 94143 USA; 18 Altos Labs, Cambridge Institute, Granta Park, Cambridgeshire, CB21 6GP UK; 19 Brigham and Women’s Hospital, Harvard Medical School, Boston, MA 02115 USA; 20 Division of Geriatric and Palliative Medicine, University of Michigan, Ann Arbor, MI 48109 USA; 21 Department of Neurology, University of Michigan, Ann Arbor, MI 48109 USA; 22 Department of Molecular and Integrative Physiology, University of Michigan, Ann Arbor, MI 48109 USA; 23 National Heart and Lung Institute, Imperial College London, London, UK

**Keywords:** Epicatechin, Halofuginone, Mitoglitazone, Survival, Genetically heterogeneous mice

## Abstract

Mice bred in 2021 were tested by the Interventions Testing Program (ITP) for possible lifespan benefits of 2BAct (2BA), dichloroacetate (DCA), Epicatechin (EPI), Forskolin (FSK), Halofuginone (HAL) and Mitoglitazone (MIT). All agents were administered in the diet ad libitum beginning at 7 months of age. In male mice, EPI increased median lifespan by ~ 5%, and HAL and MIT each increased median lifespan by ~ 9%. EPI and HAL, but not MIT, increased 90% survival. In addition to adding 3 new agents to the list of interventions identified by the ITP that extend lifespan, this report continues the strong male bias in the efficacy of life-extending drugs identified so far.

**Supplementary Information:**

The online version contains supplementary material available at 10.1007/s11357-025-01881-6.

## Introduction

The National Institute on Aging’s Interventions Testing Program (ITP) investigates compounds that are hypothesized to extend lifespan by influencing fundamental aging processes or mitigating age-related diseases. The program’s objectives are threefold: (1) to gain insights into the mechanisms of aging, (2) to provide a preclinical platform for evaluating potential therapeutics that enhance lifespan and healthspan, and (3) to assess the efficacy and safety of agents already marketed to the public [1]. Proposals for test agents are solicited annually from the research community, undergo a two-tiered review process, and are selected based on the strength of evidence supporting their potential to extend lifespan and delay aging [1]. Key strengths of the program include its rigorous and reproducible experimental design and the use of genetically diverse mouse models. Testing is conducted simultaneously at three sites: The Jackson Laboratory (TJL), the University of Michigan (UM), and the University of Texas Health Science Center at San Antonio (UT). Standardized operating procedures—covering diet formulation and distribution, animal breeding, husbandry, and monitoring—are implemented consistently across all sites [1, 2].The agents are tested in genetically heterogeneous (UM-HET3) mice to reduce strain-specific effects. Findings on the effects of all tested agents, including their impact on survival and other age-sensitive traits, are published.

Fourteen of the 60 agents previously tested by the ITP significantly increased lifespan in one or both sexes. Eight of the 14 (i.e. nordihydroguaiaretic acid (NDGA [2, 3], aspirin [2], 17-α-estradiol [3–5], Protandim [4], canagliflozin ([6, 7], astaxanthin [8], meclizine [8], and 16-hydroxyestradiol [7]) increased lifespan only in males. None of the agents tested to date have increased lifespan only in females. Three agents led to significant lifespan increases in both sexes, but with varying degrees of sex-specificity. Glycine, for example, led to small but similar increases in both sexes [9]. Acarbose effects were more dramatic in males than in females at any of the three tested doses and if started later in life, i.e. at 20 months of age [3, 4, 10]. Rapamycin, over a range of doses and at two starting ages, has had strong positive effects in both sexes [11–13], but at a given dose in chow typically leads to a larger percentage increase in female than in male mice. Interpretation of this sex-specific effect is complicated by data showing that blood levels of rapamycin are higher in female mice than in males given the same dose in chow, and it is unclear whether males and females with similar blood rapamycin levels would have different survival curves. Six of the agents that increased lifespan (NDGA, aspirin, rapamycin, 17-α-estradiol, acarbose, and canagliflozin) have been re-examined in later cohorts with different dosages and treatment durations. Of these, only aspirin did not increase lifespan, although it was only tested at higher doses [9]. Five agents have significantly shortened lifespan—all only in females: Canagliflozin [7], nevibolol [7], and 16-hydroxyestradiol [7], mycophenolic acid [8], and 4-phenylbutyrate [8]

Here we report results of six drugs in the C2021 cohort of the ITP that had not been previously tested. The rationales for testing these agents were as follows: 2BAct is a drug developed to inhibit the integrated stress response (ISR). The ISR is a key regulator of protein homeostasis. When activated during cellular stress ISR suppresses translation. There is evidence of inappropriate ISR activation during aging as well as neurodegenerative disease and suppressing ISR activation has been shown to reverse age-related declines in spatial memory and memory [14]. Treated mice performed as well as younger mice in cognitive tasks. ISR inhibition also increased the density of dendritic spines, structures essential for communication between neurons. Importantly, the cognitive benefits of ISR inhibition have been shown to persist for weeks after the drug was no longer being administered [14]. Also, suppression of ISR activation extended lifespan in C. elegans [Rosi Sl, unpublished observations; Denzel M, unpublished observations]. These data set the premise to test whether 2BAct would increase lifespan in UM-HET3 mice. Dichloroacetate (DCA) was selected based on its ability to increase pyruvate dehydrogenase complex activity [15]. In addition, DCA extended lifespan in *Drosophila* [16] and *C. elegans* [17], and showed preclinical utility in animal models of cerebral ischemia [18], vascular dementia [19], cardiac ischemia/reperfusion injury [20], hemorrhagic shock [21], and sepsis [22]. Epicatechin (EPI), a flavanol found in high concentrations in cocoa, was chosen based on preclinical trials that revealed improved vascular and skeletal muscle function and improved mitochondrial biology and angiogenesis in young and older rodents administered EPI [23]. Moreover, studies in humans suggest that EPI improves endothelial function, treadmill walking, mitochondrial function, and strength [24–26]. Forskolin (FSK) a cAMP agonist, was selected based on its multiple salutary influences on diseases and physiology, and its potential to rescue the loss of adenylate cyclase and consequently cAMP during aging of many species [27]. FSK reduced behavioral deficits and neuropathological changes in a mouse model of cerebral amyloidosis [28] and has shown efficacy against Huntington’s disease-like neurodegenerative disorders [29]. Moreover, it can exhibit antihypertensive platelet aggregation inhibition [30] and reduce glycemia and oxidative stress in rats with and without experimental diabetes [31]. Halofuginone (HAL) is a natural product-derived small molecule tRNA synthetase inhibitor that limits inflammatory damage in vivo by activating a metabolic sensor pathway called the amino acid restriction response (AAR) [32], an arm of the ISR [33]. These properties of HAL are reminiscent of numerous studies showing that amino acid restriction can extend lifespan [34]. Mitoglitazone (MIT) is a second generation PPAR_y_-sparing thiazolidinedione [35, 36]. Preclinical and clinical studies have shown that this compound, which has minimal affinity for PPAR_y_ and works primarily by modulating the mitochondrial pyruvate carrier (MPC), mimics the insulin sensitizing pharmacology of pioglitazone without the liability of direct transcriptional activators. MIT has completed successful phase 2 clinical trials in both type 2 diabetes [37] and in non-diabetic subjects with Alzheimer’s disease [38]. MIT was chosen because of its unique action as an insulin sensitizer and the spectrum of beneficial effects of insulin sensitizers on diseases of aging, as well as the growing understanding of how the second-generation compounds reprogram metabolism to protect mitochondrial function through the MPC [36, 39].

## Results

Since its inception in 2004 the ITP has used a standard analytical protocol, in which each tested drug is compared to male and female controls by site-stratified log-rank tests using data pooled from all three test sites. Reports include summary statistics, i.e. median and 90th percentile ages, for the pooled-site data for each drug for each sex. The Wang/Allison statistic [40], a Fisher exact test on proportions surviving at the 90th percentile, is used as an index for a drug's potential to support exceptionally long survival. Each ITP survival study also reports the corresponding statistics and p-values for each site considered separately. Although these tests have less statistical power than the pooled-site data, they provide useful insights into possible site-specific heterogeneity in drug effects.

**Fig. 1:**
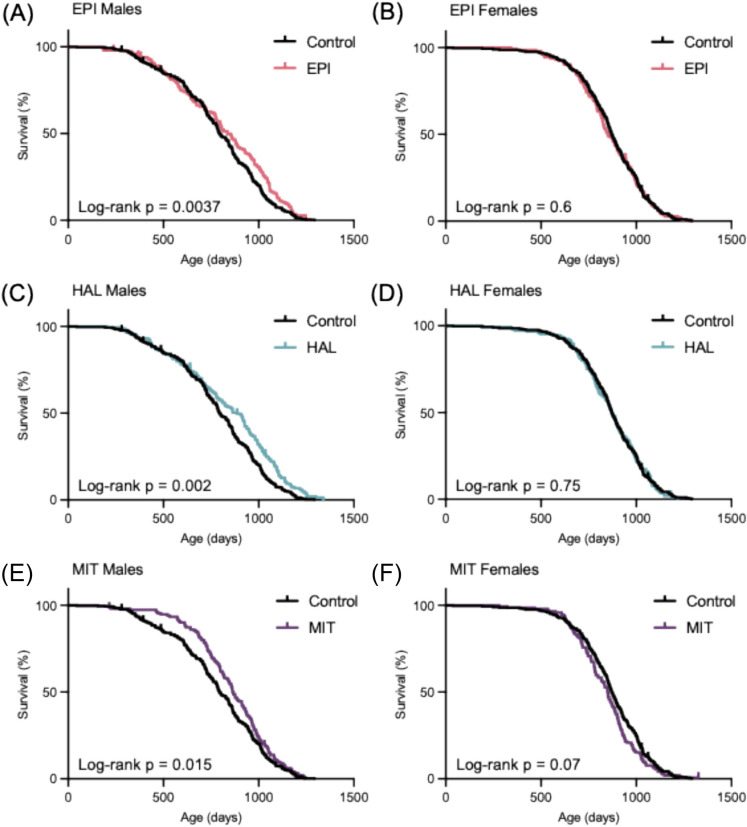

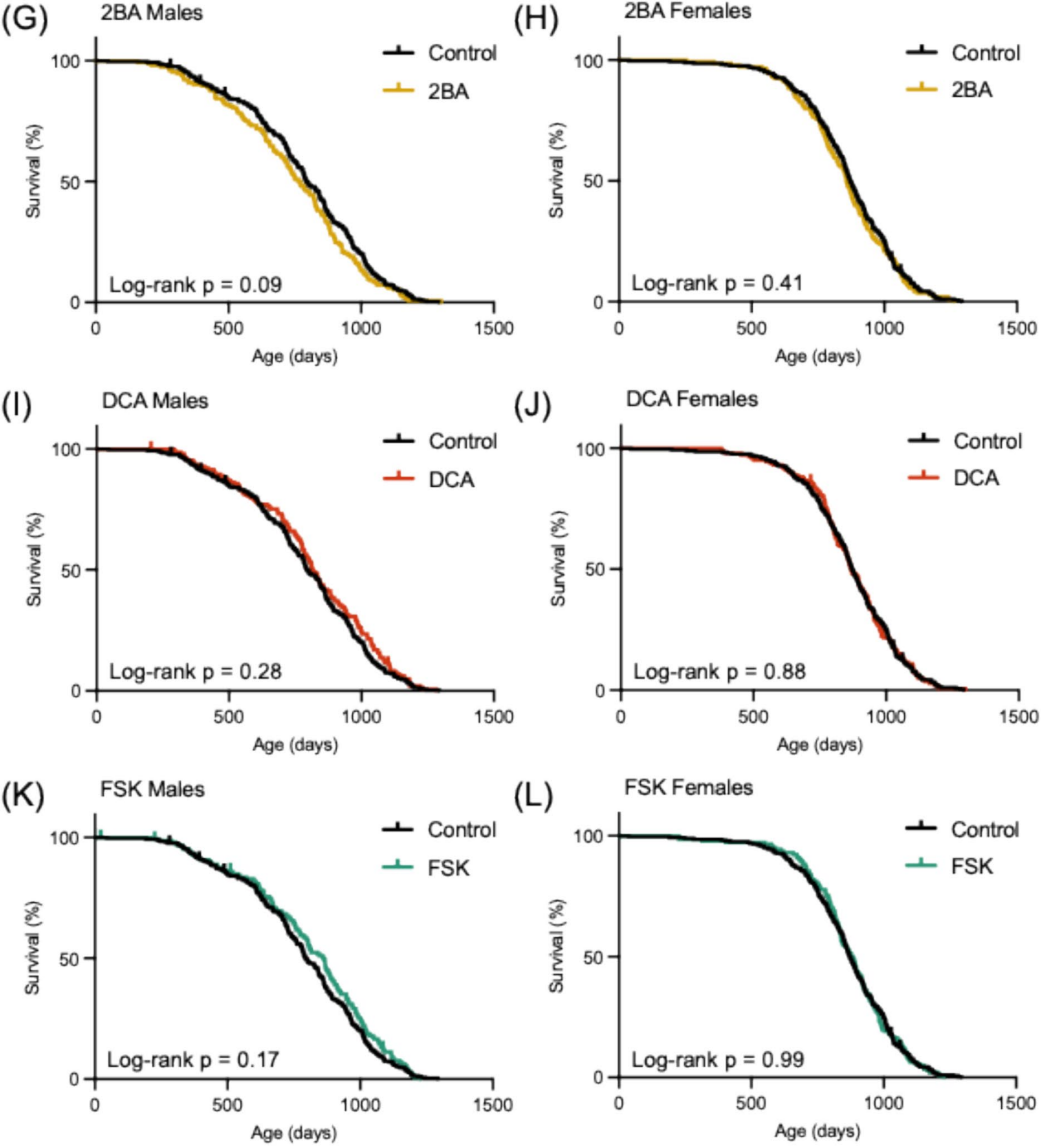
Kaplan–Meier survival plots. Agents with significant effects (EPI, HAL and MIT) are presented first, alphabetically, followed by agents with no effect (2BA, DCA and FSK) also alphabetically in males (left) and females (right). Data are pooled across the three ITP sites. Panels **A** and **B** show results for EPI, **C** and **D** for HAL, **E** and **F** for MIT, **G** and **H** for 2BA, **I** and **J** for DCA, **K** and **L** for FSK

Table 1 presents the results for data pooled across the three sites: median, log-rank p-value (stratified by site), and 90th percentile age for each drug and for each sex, with change scores compared to sex-matched control mice, along with the site-stratified Wang/Allison p-value. Tables S1a and S1b show the same statistics for each site presented separately. Figure 1 provides Kaplan–Meier plots for effects of interventions pooled across sites in both sexes. Figure S1 provides Kaplan–Meier plots for effects of interventions at each site separately.

**Table 1:** Life table statistics for mice pooled across all three test sites

Group	*n*	Median	%Change Median	Log-rank	p90	%Change p90	Wang-Allison
Males							
Cont_21	298	794			1069		
2BA	153	767	−3.4	0.09	1024	−4.2	0.42
DCA	152	814	2.5	0.28	1105	3.4	0.25
EPI	141	836	5.3	0.037	1135	6.2	0.01
FSK	150	860	8.3	0.17	1117	4.4	0.32
HAL	154	864	8.8	0.002	1143	6.9	0.01
MIT	149	863	8.7	0.015	1100	2.9	0.19
Females							
Cont_21	286	870			1079		
2BA	143	857	−1.5	0.41	1070	−0.8	0.50
DCA	142	866	−0.5	0.88	1099	1.9	0.86
EPI	143	851	−2.2	0.6	1081	0.2	0.87
FSK	143	877	0.8	0.99	1084	0.5	0.87
HAL	143	872	0.2	0.75	1076	−0.3	1.00
MIT	144	850	−2.3	0.07	1037	−3.9	0.19

Agents are presented alphabetically, p90 is 90th percentile age. *n*, median, p90, and Wang/Allison calculations exclude mice removed for fighting and other causes, for which the endpoint (death or euthanasia when moribund) did not occur. Log-rank test includes all mice entered into the study. Medians and p90 are shown in days. Percent change for median and p90 are calculated with respect to the median and p90 values for the pooled controls; they are not calculated as the mean value of site-specific median percent changes. p-Values are not adjusted for multiple comparisons

EPI, initiated at 7 months, increased median lifespan of males by ~ 5% (*p* = 0.04) and 90% survival by ~ 6% but had no effect on either statistic in females for data pooled across sites (Figs. 1A and 1B, Table 1). EPI trended toward increased median lifespan of males at all 3 sites, but the effect did not reach statistical significance at any one site (Figure S1A, Table S1b).

HAL, starting at 7 months, resulted in a ~ 9% increase in median lifespan in males (*p* = 0.002) but had no effect in females for data pooled or analyzed by site (Fig. 1, Table 1, Figure S1, Table S1). HAL increased median lifespan of males at all 3 sites, but the effect only reached statistical significance at UM. (Figure S1, Table S1) HAL increased 90% survival in males by ~ 7% for pooled data (*p* = 0.01) and trended toward increased survival at each site, but significantly only at UT (*p* = 0.01). HAL had no effect on 90% survival in females either with data pooled or analyzed by site (Table 1, Table S1).

MIT, begun at 7 months, resulted in a ~ 9% increase in median lifespan in males (*p* = 0.015) (Fig. 1, Table 1). MIT increased median lifespan of males at all 3 sites, but the effect only reached statistical significance at TJL (Figure S1, Table S1). There was a trend for decreased median lifespan of females for data pooled across sites, (*p* = 0.07) and at UM (*p* = 0.01) but no effect at TJL or UT. MIT did not affect 90% survival in either sex (Table 1).

The three other compounds, 2BA, DCA, and FSK, had no significant effect on male or female lifespan as measured by the log-rank and Wang-Allison tests (Fig. 1, Table 1). We note, however, that FSK increased median survival of males by ~ 8%, although this did not reach statistical significance. It is possible that testing FSK at other doses might produce more impressive effects.

**Fig. 2:**
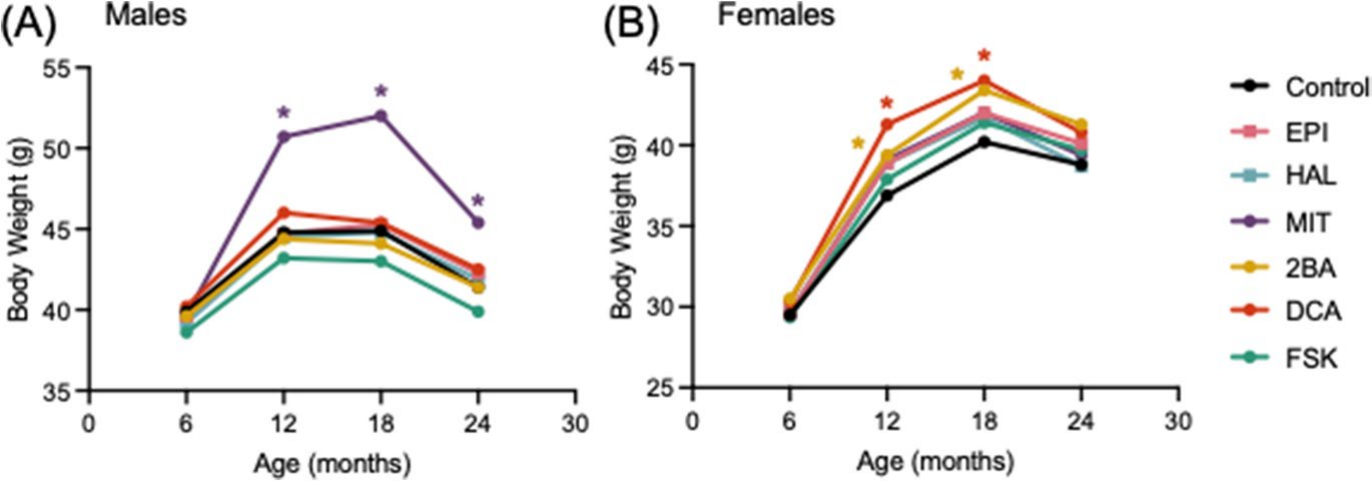
Mean body weights of treated and control mice, separated by sex. Asterisks indicate significant differences from untreated controls of the same sex and age, determined by Sidak post-hoc test following one-way ANOVAs. Males are shown in Panel **A** and females in Panel **B**

Body weights at ages 6, 12, 18 and 24 months are shown in Fig. 2 for each combination of sex and treatment group. In males, of the six agents only MIT affected body weight, increasing it relative to controls at 12, 18 and 24 months. In females, both DCA and 2BA increased body weights at 12 and 18 months.

## Discussion

During the last two decades, the ITP has identified many compounds that extend lifespan in genetically heterogeneous mice. This report adds three new agents to that list: EPI, HAL and MIT. Throughout the course of the ITP, most of the interventions have been more efficacious in males, and in many cases only efficacious in males. EPI, HAL and MIT continue this trend. To date, only one compound, rapamycin, appears to be more effective in females, although this sex disparity may reflect higher blood levels in rapamycin-treated females.

EPI, a flavanol found in cocoa, significantly increased median lifespan of males by ~ 5% and increased 90% survival by ~ 6%, but had no effect in females. The 90% survival statistic is underscored by the Kaplan Meier survival plot which shows there was no increase in survival of the population until the latter half of the lifespan, suggesting differential drug efficacy with advancing age. Through what mechanisms may EPI increase later life survival? One possibility is through increasing mitochondrial function and angiogenesis [23]. A preclinical study treating rats with EPI increased skeletal muscle mitochondrial biogenesis and mitochondrial function, measured by increases in PGC-1α, mitochondrial cristae quantity and capillary density [23]. In mice, EPI decreased skeletal myostatin, which promotes muscle wasting, and increased follistatin, which blocks myostatin’s action, thereby increasing muscle growth [24]. Human studies treating diabetic, as well as healthy young and older individuals with EPI increased endothelial function, treadmill walking, mitochondrial function and strength as well as increasing muscle mass [24–26], Epicatechin induces eNOS activation via Ser1177 and Ser633 phosphorylation and Thr495 dephosphorylation [41], Other studies show that AMPK phosphorylation of eNOS at Ser633 is a functional signaling event for NO bioavailability in endothelin cells [42]. NO has multiple beneficial effects, including increased GLUT4 [43]. These findings coupled with the lifespan benefits and the feasibility of EPI as a nutritional supplement calls for a more comprehensive study of the pharmacodynamics of EPI to probe the limits to functional improvement and lifespan extension by EPI treatment.

HAL increased median survival by ~ 9%, again only in males, and also increased their 90% survival by ~ 7% relative to controls. Like EPI, HAL had no effect on survival until after midlife. HAL and EPI are among the few male-specific agents found by the ITP to affect survival late in life. Most male-specific drugs tested by the ITP affect median survival but fail to increase 90% survival, a measure of late life efficacy. Canagloflozin and 17α-E2 are the only other male-specific agents that increase 90% survival.

HAL is a natural product-derived small molecule tRNA synthetase inhibitor which limits inflammatory damage in vivo by activating a metabolic sensor pathway called the amino acid restriction response (AAR) [32]. The AAR also increases autophagy and is anti-fibrotic [44]. Like the mTORC1 (mechanistic target of rapamycin complex 1) signaling pathway, which is inhibited by rapamycin, the AAR pathway is an amino acid sensor and a major adaptation to nutrient stress [33, 45]. The hallmark of AAR pathway activation is autophosphorylation of the ISR kinase GCN2 (General Control Nonderepressible 2), which drives a cascade of downstream effectors [46] GCN2 recently has been shown to be activated on, and by the recognition of, collided ribosomes (Keller and Whitman, manuscript under revision), which occur as a result of amino acid shortfall during protein synthesis. HAL induces the AAR by competing with proline for the prolyl-tRNA synthetase active site of glutamyl- prolyl-tRNA synthetase, thus limiting the utilization of proline for protein synthesis and mimicking restriction of this nutrient [32]. The liver plays a key role in sensing amino acid availability, and increases FGF21 synthesis and secretion in response to amino acid insufficiency. Likewise, HAL robustly increases FGF21 presence in serum (Whitman and Keller, unpublished), and FGF21 has been implicated in the lifespan extending effects of several interventions [47, 48], as well as having its own health benefits [49, 50]. The anti-inflammatory activity of HAL has been demonstrated both in vitro and in vivo [51], and these therapeutic effects fit with the finding of HAL affecting survival late in life when it could suppress inflammation-driven aging processes [52–54]. Finally, the fact that HAL activates the AAR raises the possibility that it may be a substitute or mimic of amino acid restriction, which can increase lifespan [34].

MIT significantly increased median lifespan of males by ~ 9% but trended toward a slightly shortened lifespan of females. Examination of the Kaplan Meier survival plots suggests that the positive effect of MIT on survival in males declines with advancing age, consistent with the lack of an effect of the drug on 90% survival. MIT is being developed as an insulin sensitizer based on its reprogramming of mitochondrial metabolism in multiple cell types, which like first-generation insulin sensitizers such as pioglitazone reduce inflammation and alter body composition [55]. Interestingly, prepubertal castration similarly extends median lifespan of male UM-HET3 mice by 10% [56], presumably by reducing androgen secretion. This suggests one clue to explore: namely androgen suppression or androgen resistance by MIT. In one doubleblind placebo controlled study, pioglitazone reduced testosterone levels in eugonadal men with type 2 diabetes [57]. There are sex-dependent effects in mitochondrial metabolism that might include the mitochondrial pyruvate carrier (MPC), the mitochondrial target of MIT and pioglitazone. For example, prostate cancer has a dependency on elevated pyruvate metabolism which is associated with androgen-stimulated expression of a component of the MPC [58]. Exploration of potential sex-dependent metabolic changes in specific tissues is thus warranted. These efforts may uncover mechanisms shared by multiple anti-aging interventions and can lead to new testable hypotheses.

The male-specific efficacy of MIT in UM-HET3 mice is shared by Astaxanthin [8], NDGA [2, 3], 17a-estradiol [3, 4], Meclizine [8], and Protandim [4]. Determining the extent to which these drugs may have overlapping mechanisms is an important next step. The evidence that EPI and HAL are most effective in the latter half of the lifespan whereas MIT exerts its effects on survival earlier without effect late in life suggests that the mechanisms underlying the life extension by MIT may differ from those mediating the effects of EPI and HAL. It is possible, for example, that MIT might preferentially delay lethal illnesses that tend to occur in younger male mice, whereas EPI and HAL have no effect on pathology until later in the lifespan. Testing this idea would require assessment of major causes of death for these three drugs as a function of age—an expensive undertaking. Alternatively, pharmacokinetic studies could test if the differences in timing of efficacy is associated with age-related differences in drug levels. For example, due to age-related effects on drug metabolism, plasma levels of MIT might fall below therapeutic levels with advancing age, and plasma levels of EPI and HAL may increase to effective levels only at later ages. For example, canagloflozin levels are several fold higher in older compared to younger UM-HET3 mice [6].

Although the ITP does not have the resources to conduct studies to uncover the mechanisms underlying the life extending actions of agents, it has a bank of tissues collected and frozen from treated and control mice at 22 months that are available to investigators with an interest in addressing mechanistic mechanisms or to assess whether age-related traits are also modified by the agents. In addition, the ITP and colleagues are genotyping DNA from all animals, and two studies [59, 60] have identified 59 genetic loci that modulate mortality rates as a function of age, sex and body weight. Both studies have nominated and tested candidate genes for several loci and current work is focused on gene-by-drug interaction effects.

In summary, three of six compounds significantly increased survival, but only in males. Notably, two had stronger effects in the latter half of life when the deleterious effects of aging are increasing exponentially. Although the effects of these agents on survival were modest, it is important to note that only one dose was examined, leaving open the possibility of greater effect size using different doses for each of these three agents, as well as for FSK whose suggestive beneficial results did not achieve significance at the dose tested.

## Supplementary Information

Below is the link to the electronic supplementary material.

ESM 1 (DOCX 605 KB)

ESM (DOCX 34.0 KB)

## Data Availability

The data that support the findings of this study will be openly available at The Jackson Laboratory Mouse Phenome Database at https://phenome.jax.org/search?searchterm=ITP, once the paper has been published.

## Appendix Methods

### Animals

UM-HET3 mice were produced at each of the three test sites as previously described in detail [1–3]. UM-HET3 mice are offspring from the cByB6F1 x C3D2F1 cross, so they are at least as heterogeneous genetically as wild populations, even though they are all siblings, so the population is reproducible. The mothers of the test mice were CByB6F1/J, JAX stock #100,009, whose female parents are BALB/cByJ and whose male parents are C57BL/6 J. The fathers of the test mice were C3D2F1/J, JAX stock #100,004, whose mothers are C3H/HeJ and whose fathers are DBA/2 J. For breeding cages, each site used Purina 5008 mouse chow. Mice were weaned onto the repelleted Purina 5LG6 control diet (9GGF, catalog!1,817,652–209, TestDiet, Richmond, IN.

Mice were housed as previously described (e.g., [3] in plastic cages with metal tops, using ¼ inch corn-cob bedding (Bed O'Cobs, produced by The Andersons, PO Box 114, Maumee, Ohio). Each cage was supplied with a nestlet for nest building. Mice were given free access to water, acidified (pH 2.5—2.7) by addition of hydrochloric acid, using water bottles rather than an automated watering system. Mice were housed in ventilated cages and were transferred to fresh cages every 14 days. Temperature was maintained within the range of 21 °C to 23 °C.

At the age of 42 days, each cage was assigned to a control or test group by use of a random number table. Each mouse was then briefly anesthetized by isoflurane inhalation administered either by nose cone or by an instrument designed for small animal anesthesia and a radio-frequency identification chip was implanted by sterile syringe beneath the dorsal skin between the shoulder blades, after which the wound was closed by a drop of superglue (Loctite gel, purchased locally, or Nexaband S/C, purchased from Abbott Laboratories, North Chicago, IL). UM and UT used chips purchased from AVID Microchip ID Systems (Catalog AVID3002; Folsom, LA); TJL used chips purchased from Locus Technology (Manchester, MD; catalog 1D-100A). A portion of the distal tail (1 cm) was taken and frozen for later analysis of DNA polymorphisms, after which the mouse was permitted to awaken from the anesthesia. The duration of anesthesia was approximately 1 to 2 min.

Mice received Purina 5LG6 control diet until treatments were begun. Starting at various ages as listed in the results section, mice in the treatment groups received Purina 5LG6 containing the additives, at all three sites, and mice in the control group received Purina 5LG6 without additives.

The methods utilized for health monitoring have been previously detailed [13]. In summary, each of the three colonies is assessed quarterly for infectious agents, including pinworms. All such tests were negative throughout the entire study period.

### Control and Experimental Diets

All diets were prepared by Test Diet, Inc., a division of Purina Mills (Richmond, IN). Purina 5LG6 mouse chow containing each of the test substances, and batches of control diet, were prepared at intervals of approximately 4 months. Each batch of food was shipped at the same time to each of the three test sites. Samples of diet from each batch was tested for recovery (% of compound ppm added to the diet). Detailed protocols for measuring compounds are provided in Appendix I (Supplementary Information). Table S2 lists the average recovery of each compound, averaged across the batches tested. Young adult female and male test mice were administered each diet. After 8 weeks, blood was collected and plasma concentrations of each compound were measured. Results of these measurements are shown in Table S3.

### Measurement of 2bACT in Mouse Food and Plasma using LC/MS/MS

2bACT reference standard was obtained from Aobius (Gloucester, MA). The plasma internal standard, lidocaine D10, was purchased from Milli-Pore Sigma (St. Louis, MO). All other HPLC grade reagents were purchased from Fisher Scientific (Waltham, MA). Milli-Q water was used for preparation of all solutions. A 2bACT super stock solution was prepared in methanol at a concentration of 1 mg/ml and stored in aliquots at -80 oC. A working stock solution of 2bACT was prepared daily from the super stock solution at a concentration of 100 μg/ml and used to spike the calibrators.

The LC/MS/MS system consisted of a Shimadzu SIL 20 A HT autosampler, LC-20AD pumps (2), and an AB Sciex API 4000 tandem mass spectrometer with turbo ion spray. The LC analytical column was an ACE Excel C18-AR (75 × 3.0 mm, 3 micron) purchased from Mac-Mod Analytical (Chaddsford, PA) and was maintained at 24 oC during the chromatographic runs using a Shimadzu CT-20A column oven. Mobile phase A contained 0.1% formic acid dissolved in water. Mobile phase B contained 0.1% formic acid dissolved in HPLC grade acetonitrile. The flow rate of the mobile phase was 0.4 ml/min. 2bACT and the plasma internal standard, lidocaine D10, were eluted with a gradient. The initial mobile phase was 60% B and at 1 min after injection was ramped to 100% B. From 5.0 min to 8.0 min the mobile phase was maintained at 100% B and at 8.01 min was switched immediately back to 60% B and run for 1.99 min to equilibrate the column before the next injection. 2bACT and lidocaine D10 were detected in positive mode with respective transitions of 441.3 > 237.9 Da and 245 > 96 Da.

2bACT was quantified in mouse food. Food calibrator samples were prepared by spiking food samples at concentrations of 0, 3.75, 7.5, 15, 30 and 60 ng 2bACT/mg food. 2bACT was quantified by mixing 100 mg of calibrator and unknown samples with 1 mL of HPLC grade isopropanol and then shaking for 30 min. The samples were then centrifuged for 10 min at 3,200 g. The supernatant was transferred to 1.5 ml microcentrifuge tubes and dried to residue under a warm stream of nitrogen. The residue was then redissolved with 100 µl of mobile phase B, vortexed thoroughly, and centrifuged for 2 min at 17,000 g. The samples were transferred to autosampler vials and 10 µL was injected into the LC/MS/MS. The peak area of 2bACT for each unknown sample was compared against a linear regression of the peak areas obtained by the calibration samples to quantify 2bACT. The concentration of 2bACT was expressed as ng/mg or ppm of food.

2bACT was quantified in mouse plasma. Calibrator samples were prepared daily by spiking blank plasma to achieve final concentrations of 0, 10, 50, 100, 1000, and 5000 ng/ml. Briefly, 0.1 mL of calibrator and unknown plasma samples were mixed with 10 µL of 1 µg/mL lidocaine D10 and 1 mL of HPLC grade isopropanol. The samples were vortexed vigorously and then centrifuged at 17,000 g for 5 min. The supernatants were dried to residue under a warm stream of nitrogen. The residue was then redissolved in 50 µL of mobile phase B, vortexed for 30 s, and centrifuged for 2 min at 17,000 g. The samples were transferred to injection vials and 10 µL was injected into the LC/MS/MS. The ratio of 2bACT peak area to lidocaine D10 peak area for each unknown sample was compared against a linear regression of the ratios obtained by the calibration samples to quantify 2bACT. The concentration of 2bACT was expressed as ng/mL plasma.

### Measurement of Dicholoroacetate in Mouse Food and Plasma using LC/MS/MS

Dichloroacetate (DCA) was obtained from Milli-Pore Sigma (St. Louis, MO). PEtH 16:0 18:1 D5, the plasma internal standard, was purchased from Cerilliant (Round Rock, TX). All other HPLC grade reagents were purchased from Fisher Scientific (Waltham, MA). Milli-Q water was used for preparation of all solutions.

A DCA super stock solution was prepared in methanol at a concentration of 1 mg/ml and stored in aliquots at -80 oC. A working stock solution of DCA was prepared daily from the super stock solution at a concentration of 100 μg/ml and used to spike the calibrators.

The LC/MS/MS system consisted of a Shimadzu SIL-40C XR autosampler, LC-40B XR pumps (2), and a Shimadzu 8045 tandem mass spectrometer. The LC analytical column was a Halo Penta-HILIC (100 × 2.1 mm, 2.7 micron) purchased from Mac-Mod Analytical (Chaddsford, PA) and was maintained at 40 °C during the chromatographic runs using a Shimadzu CTO-40S column oven. The isocratic mobile phase contained 40% 2 mM ammonium formate with 60% acetonitrile. The flow rate of the mobile phase was 0.2 ml/min. The analytical run was 6 min. DCA and plasma internal standard, PEtH 16:0 18:1 D5, were detected in negative mode with respective transitions of 127.0 > 82.9 Da and 706.6 > 281.3 Da.

DCA was quantified in mouse food. Food calibrator samples were prepared by spiking food samples at concentrations of 0, 0.5, 1, 5, 10, 25, and 50 ng DCA/mg food. DCA was quantified by mixing 100 mg of calibrator and unknown samples with 1 mL of HPLC grade methanol and then shaking for 30 min. The samples were then centrifuged for 30 min at 3,200 g. The supernatant was transferred to 1.5 ml microcentrifuge tubes and centrifuged for 3 min at 17,000 g. The samples were transferred to autosampler vials and 10 µL was injected into the LC/MS/MS. The peak area of DCA for each unknown sample was compared against a linear regression of the peak areas obtained by the calibration samples to quantify DCA. The concentration of DCA was expressed as ng/mg or ppm of food.

DCA was quantified in mouse plasma. Calibrator samples were prepared daily by spiking blank plasma to achieve final concentrations of 0, 1, 2.5, 5, 10, 50, 100, and 500 ng/ml. Briefly, 0.1 mL of calibrator and unknown plasma samples were mixed with 10 µL of 1 µg/mL PEtH 16:0 18:1 D5 and 1 mL of HPLC grade isopropanol. The samples were vortexed vigorously, shaken for 30 min, and then centrifuged 30 min at 3,200 g. The supernatants were transferred to a clean 1.5 ml microcentrifuge tube and dried to residue under a warm stream of nitrogen. The residue was then redissolved in 50 µL of mobile phase, vortexed for 30 s, and centrifuged for 3 min at 17,000 g. The samples were transferred to injection vials and 10 µL was injected into the LC/MS/MS. The ratio of DCA peak area to PEtH 16:0 18:1 D5 peak area for each unknown sample was compared against a linear regression of the ratios obtained by the calibration samples to quantify DCA. The concentration of DCA was expressed as ng/mL plasma.

### Measurement of epicatechin in Mouse Food and Plasma using LC/MS/MS

Standards of epicatechin and lidocaine D10, the plasma internal standard, were purchased from Milli-Pore Sigma (St. Louis, MO). All other HPLC grade reagents were purchased from Fisher Scientific (Waltham, MA). Milli-Q water was used for preparation of all solutions.

An epicatechin super stock solution was prepared in methanol at a concentration of 1 mg/ml and stored in aliquots at -80 oC. A working stock solution of epicatechin was prepared daily from the super stock solution at a concentration of 100 μg/ml and used to spike the calibrators.

The LC/MS/MS system consisted of a Shimadzu SIL 20 A HT autosampler, LC-20AD pumps (2), and an AB Sciex API 3200 tandem mass spectrometer with turbo ion spray. The LC analytical column was an ACE Excel C18-PFP (75 × 3.0 mm, 3 micron) purchased from Mac-Mod Analytical (Chaddsford, PA) and was maintained at 24 °C during the chromatographic runs using a Shimadzu CT-20A column oven. Mobile phase A contained 0.1% formic acid dissolved in water. Mobile phase B contained 0.1% formic acid dissolved in HPLC grade acetonitrile. The flow rate of the mobile phase was 0.4 ml/min. epicatechin and the plasma internal standard, lidocaine D10, were eluted with a gradient. The initial mobile phase was 25% B and at 1 min after injection was ramped to 100% B. From 6.0 min to 8.0 min the mobile phase was maintained at 100% B and at 8.01 min was switched immediately back to 25% B and run for 1.99 min to equilibrate the column before the next injection. epicatechin and lidocaine D10 were detected in positive mode with respective transitions of 291.2 > 139.2 Da and 245 > 96 Da.

Epicatechin was quantified in mouse food. Food calibrator samples were prepared by spiking food samples at concentrations of 0, 75, 150, 300, 600, and 1200 ng epicatechin/mg food. epicatechin was quantified by mixing 100 mg of calibrator and unknown samples with 1 mL of HPLC grade methanol and then shaking for 30 min. The samples were then centrifuged for 10 min at 3,200 g. The supernatant was transferred to 1.5 ml microcentrifuge tubes and centrifuged for 2 min at 17,000 g. The samples were transferred to autosampler vials and 10 µL was injected into the LC/MS/MS. The peak area of epicatechin for each unknown sample was compared against a linear regression of the peak areas obtained by the calibration samples to quantify epicatechin. The concentration of epicatechin was expressed as ng/mg or ppm of food.

Epicatechin was quantified in mouse plasma. Calibrator samples were prepared daily by spiking blank plasma to achieve final concentrations of 0, 10, 50, 100, and 500 ng/ml. Briefly, 0.1 mL of calibrator and unknown plasma samples were mixed with 10 µL of 1 µg/mL lidocaine D10 and 1 mL of HPLC grade isopropanol. The samples were vortexed vigorously and then centrifuged at 17,000 g for 5 min. The supernatants were dried to residue under a warm stream of nitrogen. The residue was then redissolved in 50 µL of mobile phase B, vortexed for 30 s, and centrifuged for 2 min at 17,000 g. The samples were transferred to injection vials and 10 µL was injected into the LC/MS/MS. The ratio of epicatechin peak area to lidocaine D10 peak area for each unknown sample was compared against a linear regression of the ratios obtained by the calibration samples to quantify epicatechin. The concentration of epicatechin was expressed as ng/mL plasma.

### Measurement of Forskolin in Mouse Food and Plasma using LC/MS/MS

Forskolin was obtained from Adooq Biosciences (Irvine, CA). Quercetin, the plasma internal standard, was purchased from Cayman Chemical (Ann Arbor, MI). All other HPLC grade reagents were purchased from Fisher Scientific (Waltham, MA). Milli-Q water was used for preparation of all solutions.

A forskolin super stock solution was prepared in methanol at a concentration of 1 mg/ml and stored in aliquots at -80 oC. A working stock solution of forskolin was prepared daily from the super stock solution at a concentration of 100 μg/ml and used to spike the calibrators.

The LC/MS/MS system consisted of a Shimadzu SIL-40C XR autosampler, LC-40B XR pumps (2), and a Shimadzu 8045 tandem mass spectrometer. The LC analytical column was an ACE Excel C18 (75 × 3.0 mm, 5 micron) purchased from Mac-Mod Analytical (Chaddsford, PA) and was maintained at 40 °C during the chromatographic runs using a Shimadzu CTO-40S column oven. Mobile phase A contained 40% 2 mM ammonium formate, 60% HPLC grade acetonitrile. Mobile phase B contained 100% HPLC grade isopropanol. The flow rate of the mobile phase was 0.5 ml/min. Forskolin and the plasma internal standard, quercetin, were eluted with a gradient. The initial mobile phase was 10% B and at 1 min after injection was ramped to 100% B. From 5.0 min to 8.0 min the mobile phase was maintained at 100% B and at 8.1 min was switched immediately back to 10% B and run for 1.9 min to equilibrate the column before the next injection. Forskolin and quercetin were detected in negative mode with respective transitions of 409 > 59.1 Da and 301.0 > 151.2 Da.

Forskolin was quantified in mouse food. Food calibrator samples were prepared by spiking food samples at concentrations of 0, 0.625, 1.25, 2.5, 5, and 10 ng forskolin/mg food. Forskolin was quantified by mixing 100 mg of calibrator and unknown samples with 1 mL of HPLC grade acetonitrile and then shaking for 30 min. The samples were then centrifuged for 10 min at 3,200 g. The supernatant was transferred to 1.5 ml microcentrifuge tubes and centrifuged for 3 min at 17,000 g. The samples were transferred to autosampler vials and 10 µL was injected into the LC/MS/MS. The peak area of forskolin for each unknown sample was compared against a linear regression of the peak areas obtained by the calibration samples to quantify forskolin. The concentration of forskolin was expressed as ng/mg or ppm of food.

Forskolin was quantified in mouse plasma. Calibrator samples were prepared daily by spiking blank plasma to achieve final concentrations of 0, 2.5, 5, 10, 50, 100, and 500 ng/ml. Briefly, 0.1 mL of calibrator and unknown plasma samples were mixed with 10 µL of 1 µg/mL quercetin and 1 mL of HPLC grade isopropanol. The samples were vortexed vigorously, shaken for 30 min, and then centrifuged at 3,200 g for 30 min. The supernatants were dried to residue under a warm stream of nitrogen. The residue was then redissolved in 50 µL of 50/50 mobile phase A/mobile phase B, vortexed for 30 s, and centrifuged for 3 min at 17,000 g. The samples were transferred to injection vials and 10 µL was injected into the LC/MS/MS. The ratio of forskolin peak area to quercetin peak area for each unknown sample was compared against a linear regression of the ratios obtained by the calibration samples to quantify forskolin. The concentration of forskolin was expressed as ng/mL plasma.

### Measurement of Halofuginone in Mouse Food and Plasma using LC/MS/MS

Standards of halofuginone (halo) and lidocaine D10, the plasma internal standard, were purchased from Milli-Pore Sigma (St. Louis, MO). All other HPLC grade reagents were purchased from Fisher Scientific (Waltham, MA). Milli-Q water was used for preparation of all solutions.

A halo super stock solution was prepared in methanol at a concentration of 1 mg/ml and stored in aliquots at -80 oC. A working stock solution of halo was prepared daily from the super stock solution at a concentration of 100 μg/ml and used to spike the calibrators.

The LC/MS/MS system consisted of a Shimadzu SIL-40C XR autosampler, LC-40B XR pumps (2), and a Shimadzu 8045 mass spectrometer. The LC analytical column was an ACE Excel C18-AR (75 × 3.0 mm, 3 micron) purchased from Mac-Mod Analytical (Chaddsford, PA) and was maintained at 40 °C during the chromatographic runs using a Shimadzu CTO-40S column oven. Mobile phase A contained 0.1% formic acid dissolved in water. Mobile phase B contained 0.1% formic acid dissolved in HPLC grade acetonitrile. The flow rate of the mobile phase was 0.4 ml/min. Halo and the plasma internal standard, lidocaine D10, were eluted with a gradient. The initial mobile phase was 15% B and at 1 min after injection was ramped to 100% B. From 4.0 min to 8.0 min the mobile phase was maintained at 100% B and at 8. 1 min was switched immediately back to 15% B and run for 1.9 min to equilibrate the column before the next injection. Halo and lidocaine D10 were detected in positive mode with respective transitions of 416 > 100.1 Da and 245 > 96 Da.

Halo was quantified in mouse food. Food calibrator samples were prepared by spiking food samples at concentrations of 0, 0.075, 0.15, 0.3, 0.6, and 1.2 ng halo/mg food. Halo was quantified by mixing 100 mg of calibrator and unknown samples with 1 mL of HPLC grade isopropanol and then shaking for 30 min. The samples were then centrifuged for 10 min at 3,200 g. The supernatant was transferred to 1.5 ml microcentrifuge tubes and centrifuged for 2 min at 17,000 g. The samples were transferred to autosampler vials and 10 µL was injected into the LC/MS/MS. The peak area of halo for each unknown sample was compared against a linear regression of the peak areas obtained by the calibration samples to quantify halo. The concentration of halo was expressed as ng/mg or ppm of food.

Halo was quantified in mouse plasma. Calibrator samples were prepared daily by spiking blank plasma to achieve final concentrations of 0, 5, 10, 50, and 100 ng/ml. Briefly, 0.1 mL of calibrator and unknown plasma samples were mixed with 10 µL of 1 µg/mL lidocaine D10 and 1 mL of HPLC grade methanol. The samples were vortexed vigorously and then centrifuged at 17,000 g for 5 min. The supernatants were dried to residue under a warm stream of nitrogen. The residue was then redissolved in 50 µL of mobile phase B, vortexed for 30 s, and centrifuged for 2 min at 17,000 g. The samples were transferred to injection vials and 10 µL was injected into the LC/MS/MS. The ratio of halo peak area to lidocaine D10 peak area for each unknown sample was compared against a linear regression of the ratios obtained by the calibration samples to quantify halo. The concentration of halo was expressed as ng/mL plasma.

### Measurement of Mitoglitazone (MSDC-160) in Mouse Food and Plasma using LC/MS/MS

Standards of mitoglitazone (MSDC-60) and lidocaine D10, the plasma internal standard, were purchased from Milli-Pore Sigma (St. Louis, MO). All other HPLC grade reagents were purchased from Fisher Scientific (Waltham, MA). Milli-Q water was used for preparation of all solutions.

A MSDC-60 super stock solution was prepared in methanol at a concentration of 1 mg/ml and stored in aliquots at -80 oC. A working stock solution of MSDC-60 was prepared daily from the super stock solution at a concentration of 100 μg/ml and used to spike the calibrators.

The LC/MS/MS system consisted of a Shimadzu SIL 20 A HT autosampler, LC-20AD pumps (2), and an AB Sciex API 4000 tandem mass spectrometer with turbo ion spray. The LC analytical column was an ACE Excel C18-AR (75 × 3.0 mm, 3 micron) purchased from Mac-Mod Analytical (Chaddsford, PA) and was maintained at 24 °C during the chromatographic runs using a Shimadzu CT-20A column oven. Mobile phase A contained 0.1% formic acid dissolved in water. Mobile phase B contained 0.1% formic acid dissolved in HPLC grade acetonitrile. The flow rate of the mobile phase was 0.4 ml/min. MSDC-60 and the plasma internal standard, lidocaine D10, were eluted with a gradient. The initial mobile phase was 15% B and at 1 min after injection was ramped to 100% B. From 4.0 min to 8.0 min the mobile phase was maintained at 100% B and at 8.01 min was switched immediately back to 15% B and run for 1.99 min to equilibrate the column before the next injection. MSDC-60 and lidocaine D10 were detected in positive mode with respective transitions of 371 > 149.1 Da and 245 > 96 Da.

MSDC-60 was quantified in mouse food. Food calibrator samples were prepared by spiking food samples at concentrations of 0, 37.5, 75, 150, 300, and 600 ng MSDC-60/mg food. MSDC-60 was quantified by mixing 100 mg of calibrator and unknown samples with 1 mL of HPLC grade isopropanol and then shaking for 30 min. The samples were then centrifuged for 10 min at 3,200 g. The supernatant was transferred to 1.5 ml microcentrifuge tubes and centrifuged for 2 min at 17,000 g. All the samples were then diluted 100 × in isopropanol and vortexed. The samples were transferred to autosampler vials and 10 µL was injected into the LC/MS/MS. The peak area of MSDC-60 for each unknown sample was compared against a linear regression of the peak areas obtained by the calibration samples to quantify MSDC-60. The concentration of MSDC-60 was expressed as ng/mg or ppm of food.

MSDC-160 was quantified in mouse plasma. Calibrator samples were prepared daily by spiking blank plasma to achieve final concentrations of 0, 5, 10, 50, 100, and 500 ng/ml. Briefly, 0.1 mL of calibrator and unknown plasma samples were mixed with 10 µL of 1 µg/mL lidocaine D10 and 1 mL of HPLC grade methanol. The samples were vortexed vigorously and then centrifuged at 17,000 g for 5 min. The supernatants were dried to residue under a warm stream of nitrogen. The residue was then redissolved in 50 µL of mobile phase B, vortexed for 30 s, and centrifuged for 2 min at 17,000 g. The samples were transferred to injection vials and 10 µL was injected into the LC/MS/MS. The ratio of MSDC-60 peak area to lidocaine D10 peak area for each unknown sample was compared against a linear regression of the ratios obtained by the calibration samples to quantify MSDC-60. The concentration of MSDC-60 was expressed as ng/mL plasma.

### Removal of Mice from the Longevity Population

As described in detail [12], mice were removed from the study because of fighting, or accidental death, typically during chip implantation, or because of chip failure, or because they were used for another experimental purpose, such as testing for blood levels of a test agent. For survival analyses, all such mice were treated as alive at the date of their removal from the protocol and lost to follow-up thereafter. These mice were not included in calculations of median longevity.

### Estimation of age at death (lifespan).

Mice were examined twice daily for signs of ill health. Mice were euthanized for humane reasons if so severely moribund that they were considered, by an experienced technician, unlikely to survive for more than an additional 48 h. A mouse was considered severely moribund if it exhibited more than one of the following clinical signs:

- inability to eat or to drink;
- severe lethargy, as indicated by reluctance to move when gently prodded with a forceps;
- severe balance or gait disturbance;
- rapid weight loss over a period of one week or more; or
- an ulcerated or bleeding tumor. The age at which a moribund mouse was euthanized was taken as the best available estimate of its natural lifespan. Mice found dead were also noted at each daily inspection.

### Statistical Methods

Standard analyses: The statistical analytical plan was specified before initiating the study and has been followed for all ITP studies. For each sex, we performed site specific and combined site analysis. We calculated the median survival for the control group as well as for each treatment group (omitting mice removed for reasons specified above). To compute the median percentage increase, we subtracted the median age in the control group from the corresponding value in the treatment group and divided the difference by median age of the control group and multiplied by 100. Using a two-sided 5% significance level, we performed the log-rank test to determine whether survival curves for mice receiving treatment differ from the survival function for control mice. Log-rank tests that pooled data across the three test sites used a method that stratifies by site. As a surrogate for “maximum” life span, we computed 90th percentile ages of both the treated and control mice. To determine which treatments led to exceptionally long life, we utilized the Wang-Allison test [39]. This is the Fisher Exact Test comparing the numbers of mice surviving in control and treatment group at the age corresponding to the 90th percentile of lifespan in the joint survival distribution. We further assessed the longevity of mice in all three sites combined using a modified version of the Wang-Allison test in the manner with which the 2 × 2 contingency table is constructed separately for each site. Basically, we report the sum of corresponding site-specific 2 × 2 tables cell entries as the combined site 2 × 2 table cell entries. This allows for information from all sites to be used in a balanced manner.
