# Supplementary material for: Extension of lifespan by epicatechin, halofuginone and mitoglitazone in male but not female genetically heterogeneous mice
Source: GeroScience. 2025 Sep 19;48(3):4139–56. doi: 10.1007/s11357-025-01881-6 (PMC13355999; doi:10.1007/s11357-025-01881-6)
Supplement: Supplementary file 1 — (DOCX 605 KB) [file 11357_2025_1881_MOESM1_ESM.docx]

Appendix C Supplementary Figure

**Figure S1 Kaplan-Meier site-specific survival plots.** Agents with significant effects using pooled data (EPI, HAL and MIT) are presented alphabetically by site, as are agents with no effect (2BA, DCA and FSK)
