# Supplementary material for: Extension of lifespan by epicatechin, halofuginone and mitoglitazone in male but not female genetically heterogeneous mice
Source: GeroScience. 2025 Sep 19;48(3):4139–56. doi: 10.1007/s11357-025-01881-6 (PMC13355999; doi:10.1007/s11357-025-01881-6)
Supplement: Supplementary file 2 — (DOCX 34.0 KB) [file 11357_2025_1881_MOESM2_ESM.docx]

Appendix B Supplementary Tables

**Table S1a: Male Survival Statistics by Test Site**

| Group | *n* | Median | %Change Median | Log-rank | p90 | %Change p90 | Wang-Allison |
| --- | --- | --- | --- | --- | --- | --- | --- |
| **Males at TJL** | |  |  |  |  |  |  |
| Cont_21 | 97 | 715 |  |  | 1048 |  |  |
| 2BA | 51 | 749 | 4.8 | 0.88 | 959 | -8.5 | 0.58 |
| DCA | 48 | 780 | 9.1 | 0.38 | 1069 | 2.0 | 0.57 |
| EPI | 42 | 800 | 11.9 | 0.06 | 1109 | 5.8 | 0.36 |
| FOR | 50 | 758 | 6.0 | 0.09 | 1138 | 8.6 | 0.15 |
| HAL | 51 | 779 | 9.0 | 0.06 | 1094 | 4.4 | 0.39 |
| MIT | 51 | 860 | 20.3 | 0.005 | 1133 | 8.1 | 0.39 |
| **Males at UM** | |  |  |  |  |  |  |
| Cont_21 | 99 | 874 |  |  | 1123 |  |  |
| 2BA | 51 | 826 | -5.5 | 0.1 | 1024 | -8.8 | 0.77 |
| DCA | 53 | 845 | -3.3 | 0.41 | 1161 | 3.4 | 0.42 |
| EPI | 48 | 880 | 0.7 | 0.32 | 1173 | 4.5 | 1.00 |
| FOR | 49 | 887 | 1.5 | 0.96 | 1079 | -3.9 | 0.39 |
| HAL | 54 | 926 | 5.9 | 0.15 | 1137 | 1.2 | 0.58 |
| MIT | 50 | 912 | 4.3 | 0.65 | 1120 | -0.3 | 1.00 |
| **Males at UT** | |  |  |  |  |  |  |
| Cont_21 | 102 | 783 |  |  | 1039 |  |  |
| 2BA | 51 | 709 | -9.5 | 0.24 | 1047 | 0.8 | 0.78 |
| DCA | 51 | 784 | 0.1 | 0.87 | 1080 | 4.3 | 0.40 |
| EPI | 51 | 805 | 2.8 | 0.43 | 1135 | 9.2 | 0.40 |
| FOR | 51 | 809 | 3.3 | 0.54 | 1117 | 7.5 | 0.16 |
| HAL | 49 | 864 | 10.3 | 0.04 | 1215 | 16.9 | 0.01 |
| MIT | 48 | 863 | 10.2 | 0.39 | 1080 | 3.9 | 0.56 |

**Table S1b: Female Survival Statistics by Test Site**

| Group | *n* | | Median | %Change Median | Log-rank | p90 | %Change p90 | Wang-Allison |
| --- | --- | --- | --- | --- | --- | --- | --- | --- |
| **Females at TJL** | | |  |  |  |  |  |  |
| Cont_21 | | 96 | 892 |  |  | 1079 |  |  |
| 2BA | | 47 | 883 | -1.0 | 0.33 | 1114 | 3.2 | 0.40 |
| DCA | | 47 | 900 | 0.9 | 0.64 | 1040 | -3.6 | 0.77 |
| EPI | | 48 | 876 | -1.8 | 0.42 | 1061 | -1.7 | 0.39 |
| FOR | | 47 | 869 | -2.6 | 0.83 | 1079 | 0.0 | 1.00 |
| HAL | | 48 | 914 | 2.5 | 0.97 | 1080 | 0.1 | 1.00 |
| MIT | | 48 | 883 | -1.0 | 0.9 | 1114 | 3.2 | 0.57 |
| **Females at UM** | | |  |  |  |  |  |  |
| Cont_21 | | 94 | 882 |  |  | 1103 |  |  |
| 2BA | | 48 | 838 | -5.0 | 0.01 | 1040 | -5.4 | 0.26 |
| DCA | | 47 | 816 | -7.5 | 0.85 | 1105 | 0.5 | 0.57 |
| EPI | | 47 | 894 | 1.4 | 0.51 | 1127 | 2.5 | 1.00 |
| FOR | | 48 | 877 | -0.6 | 0.3 | 1062 | -3.4 | 0.77 |
| HAL | | 47 | 867 | -1.7 | 0.45 | 1074 | -2.3 | 0.77 |
| MIT | | 48 | 830 | -5.9 | 0.01 | 1026 | -6.6 | 0.09 |
| **Females at UT** | | |  |  |  |  |  |  |
| Cont_21 | | 96 | 848 |  |  | 1050 |  |  |
| 2BA | | 48 | 838 | -1.2 | 0.87 | 1064 | 1.3 | 0.40 |
| DCA | | 48 | 856 | 0.9 | 0.7 | 1114 | 6.1 | 1.00 |
| EPI | | 48 | 835 | -1.5 | 0.43 | 1071 | 2.0 | 1.00 |
| FOR | | 48 | 895 | 5.5 | 0.24 | 1100 | 4.8 | 0.26 |
| HAL | | 48 | 832 | -1.9 | 0.88 | 1079 | 2.8 | 0.26 |
| MIT | | 48 | 848 | 0.0 | 0.41 | 1034 | -1.5 | 0.58 |
|  | |  |  |  |  |  |  |  |

**Table S2 Food concentrations of compounds.** Prior to initiation of each study, Pilot food was manufactured and tested prior to feeding young adult test mice for 8 weeks on the diet. Subsequently, diet was manufactured to food to mice across their lifespan. Samples of this diet was tested after 3-months of storage at 4º C. Subsequently food was tested again at the end of the lifespan study. Target concentration (in ppm) is shown, followed by measured concentration (in PPM, and as a percentage of the target concentration), and finally the coefficient of variation, expressed as a percentage of the mean (CV%) for each of these food samples. Methods for measuring each compound are described below.

| **Drug** | **Target PPM** | **Pilot Food** | | | **Lifespan Food** | | | **Food Stability (3-month)** | | | **End of Study Food** | | |
| --- | --- | --- | --- | --- | --- | --- | --- | --- | --- | --- | --- | --- | --- |
|  |  | **Actual PPM** | **% expected** | **CV %** | **Actual PPM** | **% expected** | **CV %** | **Actual PPM** | **% expected** | **CV %** | **Actual PPM** | **% expected** | **CV %** |
| **2bACT** | 30.0 | 33.4 | 111.2 | 0.8 | 25.4 | 84.8 | 3.7 | 40.6 | 135.3 | 12.8 | 81.0 | 81.0 | 3.2 |
| **DCA** | 30.0 | 15.5 | 52.0 | 6.4 | 5.9 | 19.6 | 7.3 | 1.1 | 3.7 | 2.8 |  |  |  |
| **Epicatechin** | 600.0 | 138.0 | 23.0 | 3.6 | 207.3 | 34.5 | 2.8 | 201.0 | 33.5 | 2.5 | 409.3 | 68.2 | 1.7 |
| **Forskolin** | 5.0 | 0.9 | 18.7 | 0.2 | 3.2 | 64.7 | 1.7 | 2.3 | 45.4 | 30.3 | 5.4 | 107.8 | 2.0 |
| **Halofuginone** | 0.6 | 0.2 | 26.2 | 2.8 | 0.2 | 36.4 | 8.1 | 0.1 | 17.8 | 5.5 | 0.1 | 22.7 | 4.5 |
| **Mitoglitazone** | 300.0 | 39.0 | 13.0 | 2.7 | 137.6 | 45.9 | 8.7 | 96.8 | 32.3 | 7.6 | 119.5 | 39.8 | 5.1 |

**Table S3. Plasma concentrations of compounds.** Prior to initiation of each study, young adult test mice were given the diet and after 8 weeks on the diet, plasma was collected, and compounds measured. Methods for measuring each compound are described below.

|  | **Female** | | | **Male** | | |
| --- | --- | --- | --- | --- | --- | --- |
|  | **Plasma (ng/ml)** | | | **Plasma (ng/ml)** | | |
| **Intervention** | **Min** | **Mean** | **Max** | **Min** | **Mean** | **Max** |
| **2BA** | 3220 | 4797 | 6337 | 3200 | 4197 | 5500 |
| **DCA*** | 9.5 | 9.5 | 9.5 | n/d | n/d | n/d |
| **EPI** | n/d | n/d | n/d | n/d | n/d | n/d |
| **FSK** | 1.1 | 4.8 | 13.2 | 1.2 | 3.0 | 5.0 |
| **HAL** | n/d | n/d | n/d | n/d | n/d | n/d |
| **MIT** | 5.2 | 35.0 | 82 | 17.8 | 34.0 | 74.7 |
| n/d – not detected in sample |  |  |  |  |  |  |
| * - Only one detectable result |  |  |  |  |  |  |
